# Supplementary material for: LGR5 controls extracellular matrix production by stem cells in the developing intestine
Source: EMBO Rep. 2020 May 28;21(7):e49224. doi: 10.15252/embr.201949224 (PMC7332981; doi:10.15252/embr.201949224)
Supplement: Supplementary file 1 — Expanded View Figures PDF [file EMBR-21-e49224-s001.pdf]

Expanded View Figures

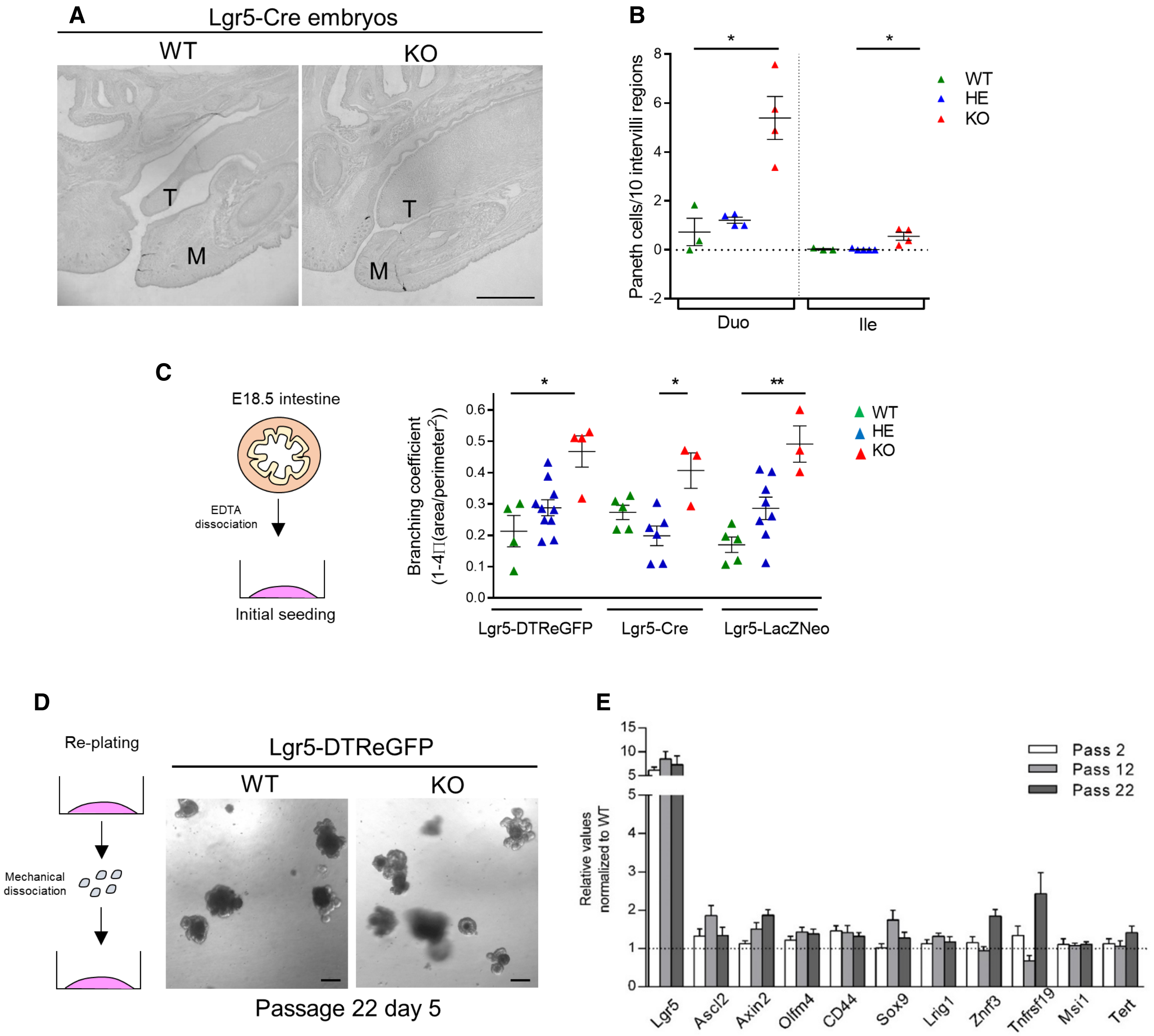

Figure EV1. Lgr5 deficiency induces early Paneth cell differentiation and stem cell expansion in the small intestine at E18.5.

A Sagittal sections of craniofacial region showing the presence of an ankyloglossia in Lgr5-null Cre embryos at E18.5 as compared to the WT littermate. The tongue (T) and mandible (M) are indicated.

B Paneth cell quantification as number of cells per 10 intervillus regions on Lgr5-Cre duodenum (Duo) and ileum (Ile). Each symbol indicates the value for a given embryo.

C Quantification showing organoid complexity ex vivo measured by the branching coefficient for wild-type (WT), heterozygous (HE), and KO organoids from 3 different mouse strains: Lgr5-DTReGFP, Lgr5-Cre, and Lgr5-LacZNeo at day 6 of culture upon initial seeding. Each symbol indicates the mean value for a given embryo.

D Representative pictures of WT and KO Lgr5-DTReGFP organoids at day 5 of passage 22.

E Gene expression analysis by qRT–PCR of the indicated stem cell markers in Lgr5-DTReGFP WT and Lgr5 KO organoid cultures, each originating from a given embryo ($n = 7$ WT and 9 KO at passage 2 and passage 12, $n = 2$ WT and 5 KO at passage 22). Values are normalized to the WTs at passage 2.

Data information: Scale bars, 1 mm (A) and 50 μm (D). Data are represented as means ± SEM. *$P < 0.05$; **$P < 0.01$ by Kruskal–Wallis test followed by Dunn's multiple comparison test (B, C).

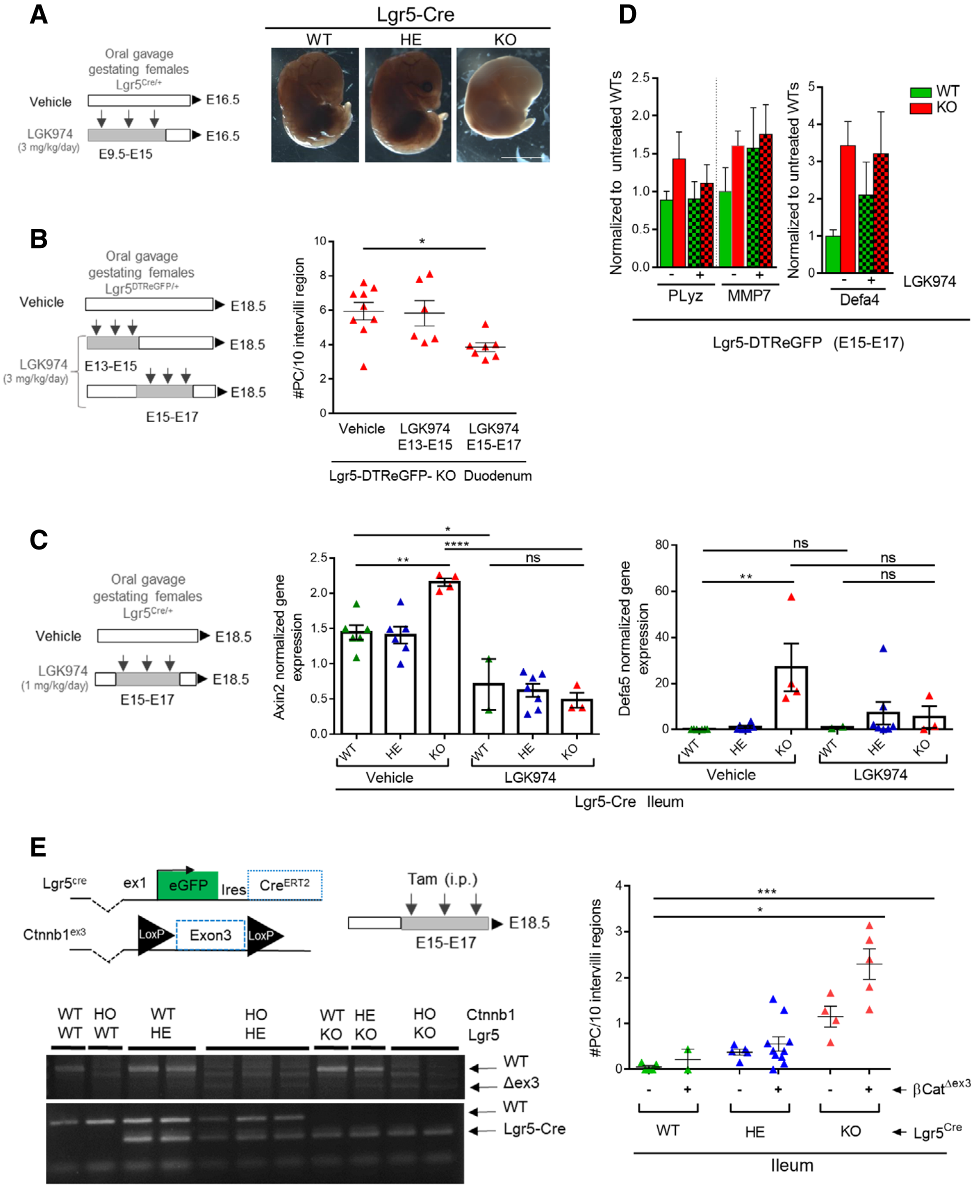

Figure EV2. In utero inhibition of Wnt activity counteracts early Paneth cell differentiation induced by Lgr5 deficiency.

A Gestating Lgr5-Cre heterozygous females were vehicle- or LGK974-treated by oral gavage between E9.5 and E15 at the indicated dose. Global morphological analysis of whole embryos was done at E16.5.

B Gestating Lgr5-DTReGFP heterozygous females were vehicle- or LGK974-treated by oral gavage between E13-E15 or E15-E17 at the indicated dose. Duodenum of treated Lgr5-DTReGFP KO embryos was analyzed at E18.5 for Paneth cell differentiation. Each symbol indicates the value for a given embryo.

C Gestating Lgr5-Cre heterozygous females were vehicle- or LGK974-treated by oral gavage between E15 and E17 at the indicated dose. Gene expression analysis of stem cell (Axin2) and Paneth differentiation (Defa5) markers was performed at E18.5 in ileums of Lgr5-Cre embryos by qRT–PCR. Each symbol indicates the value for a given embryo.

D Gene expression analysis of Paneth differentiation markers was performed at E18.5 in ileums of Lgr5-DTReGFP embryos by qRT–PCR (vehicle-treated: 7 WT and 7 KO; LGK974-treated: 3 WT and 6 KO).

E Gestating females were intra-peritoneally injected with tamoxifen between E15 and E17. Left panel: Recombination of floxed b-catenin exon 3 (Δex3) was verified by PCR on ileums of embryos. Genotypes of embryos for b-catenin (Ctnnb1) and Lgr5 loci are indicated. Right panel: Ileums of embryos were analyzed at E18.5 for Paneth cell differentiation by Lendrum staining. Each symbol indicates the value for a given embryo.

Data information: Scale bar, 500 μm. Data are represented as means ± SEM. **P* < 0.05; ***P* < 0.01; ****P* < 0.001; *****P* < 0.0001 by Kruskal–Wallis test followed by Dunn's multiple comparison test (B, E) and two-way ANOVA followed by Tukey's multiple comparison test (C, D).

 © 2020 The Authors

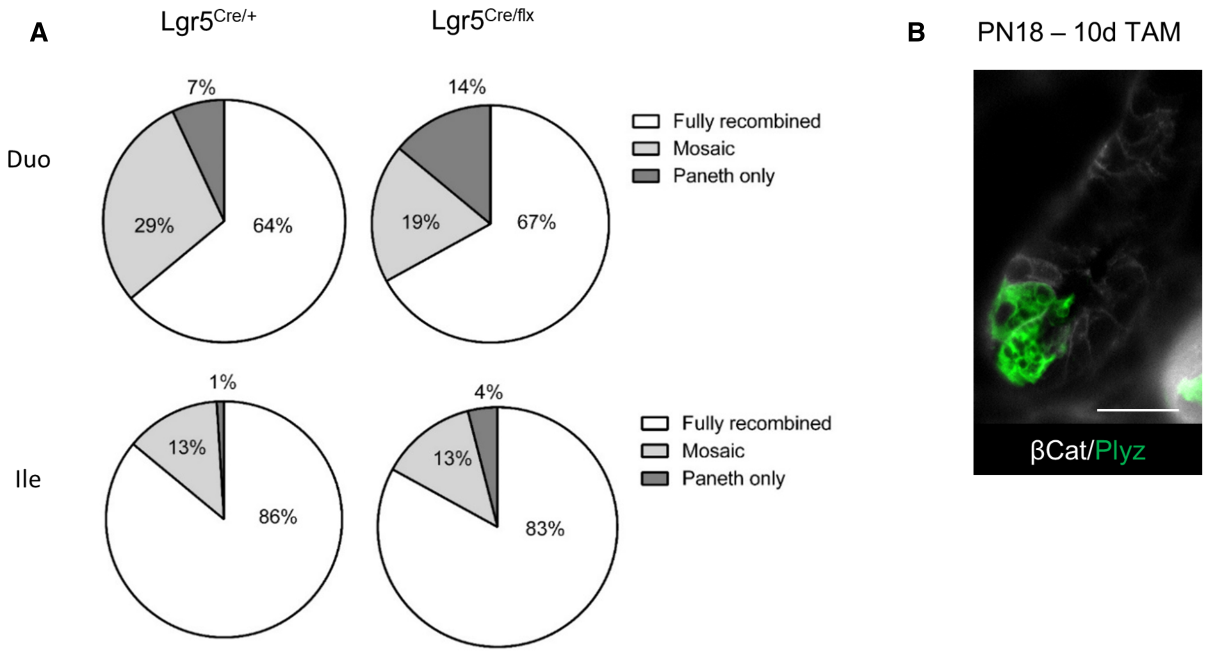

Figure EV3. Postnatal Lgr5 ablation in ISCs alters stem cell fate toward the Paneth cell lineage.

A Fate of RFP^{+ve}-traced clones in control ($Lgr5\text{-}^{Cre/+}$) and cKO ($Lgr5\text{-}^{Cre/flx}$) at postnatal day 18 (PN18) after 10 days of chase. The proportion of fully recombined and mosaic RFP^{+ve} crypt/villus units as well as RFP^{+ve} cells only labeling Paneth cells is indicated for the duodenum and ileum of controls and cKOs (*n* = 5 for each genotype).

B Representative immunofluorescence picture showing Paneth cells (Plyz marker) in co-staining with the epithelial cell membrane marker b-catenin in a control ($Lgr5^{Cre/+}$) at PN18 in ileum 10 days after tamoxifen injection. Scale bar, 50 μm.

© 2020 The Authors

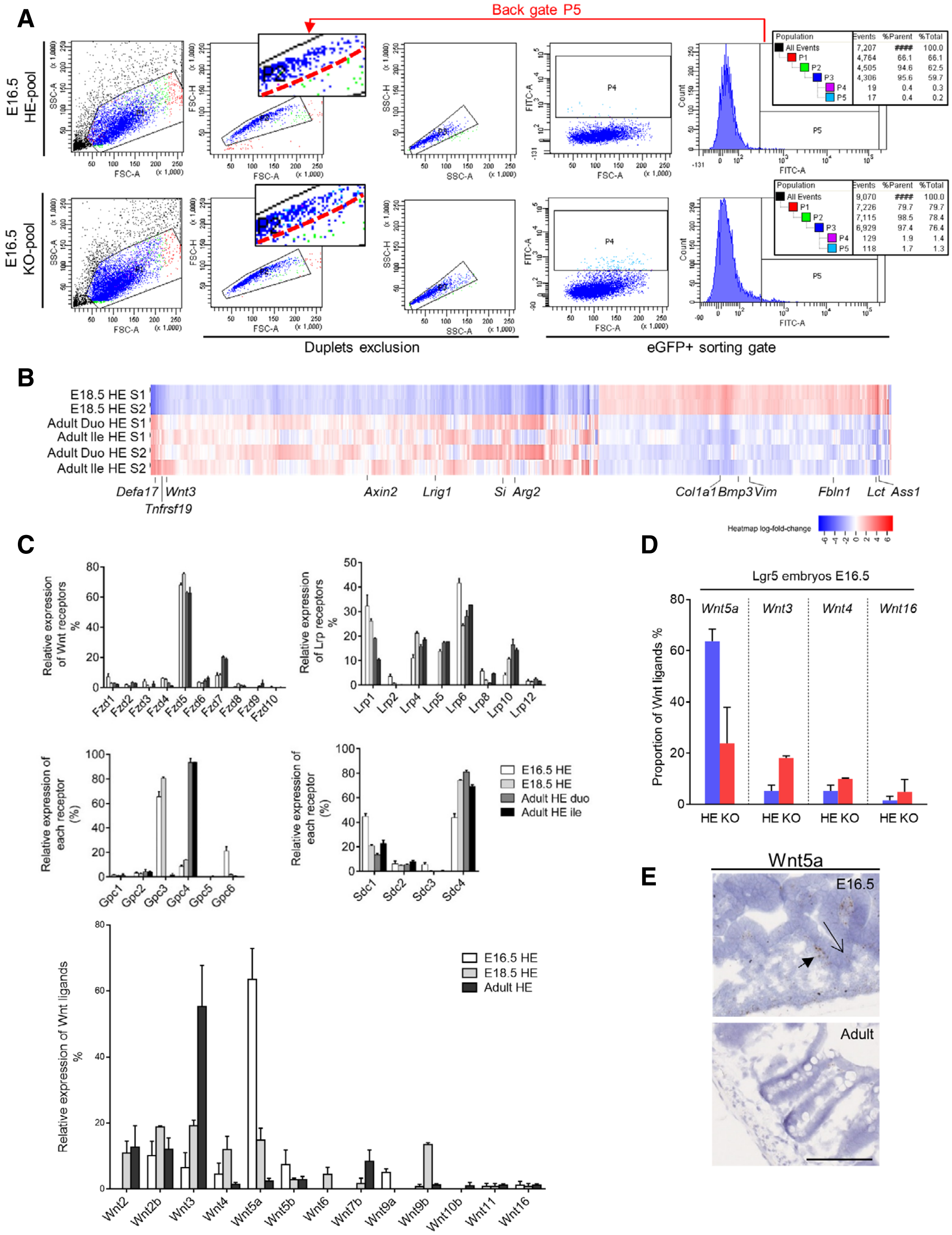

Figure EV4. Transcriptome analysis of Lgr5 ISC precursors in adults.

A Representative FACS plots showing gating strategy used for sorting experiments (Lgr5-DTReGFP E16.5). Back gate for eGFP+ sorted cells (visualized as cyan dots) shows high stringency for duplets exclusion (depicted by a red dashed line).

B ISC (eGFP^{+ve}) cells from Lgr5-DTReGFP embryonic E18.5 and adult stages were sorted by FACS and subjected to RNAseq analysis. Heatmap of differentially regulated genes (fold change × 1.5, FDR 0.1) in E18.5 HE as compared to adult ISCs from duodenum (Duo) and ileum (Ile). Selected genes are evidenced. Highlighted genes: Wnt-related genes (Wnt3, Axin2), differentiation markers (Defa17, Si), extracellular matrix-related genes (Col1a1, Vim), and metabolic-related genes (Lct, Ass1).

C Graphs showing relative expression levels of the Wnt ligands and Wnt receptors/co-receptors (Fzd, Lrp, Gpc, Sdc) at different developmental stages E16.5, E18.5, and adult HEs based on the RNAseq data.

D Graph showing relative proportion of the main Wnt ligands expressed in Lgr5 KOs and HEs at E16.5.

E Expression of the Wnt ligand Wnt5a in normal embryonic and adult small intestines by RNAscope. The arrows show Wnt5a-expressing cells localized in stroma and epithelium in E16.5 embryos.

Data information: Scale bar, 100 μm. Biological replicates for RNAseq experiments on ISC (B, C, D): Lgr5-DTReGFP E16.5 independent embryonic pools (n = 2 HE, 2 KO); Lgr5-DTReGFP E18.5 individual embryos (n = 2 HE depicted as S1 and S2 in panel B), and Lgr5-DTReGFP adult individual animals (n = 2 HE depicted as S1 and S2 in panel B).

 © 2020 The Authors

A

Gene / Tissue sample	Duo E18.5 CT (mean ± SD)	Ile E18.5 CT (mean ± SD)	E18.5 (Duo/Ile) RT neg
Rspo1	33.2 ± 0.6	31.5 ± 0.5	>36/>36
Rspo2	28.9 ± 0.6	29.7 ± 0.5	>36/>36
Rspo3	30.6 ± 0.4	29.5 ± 0.8	>36/>36
Rspo4	>35	>35	>36/>36

Gene / Tissue sample	Duo adult CT (mean ± SD)	Ile adult CT (mean ± SD)	Adult (Duo/Ile) RT neg
Rspo1	>35	>35	>36/>36
Rspo2	34.6 ± 0.1	33.1 ± 0.1	>36/>36
Rspo3	34.6 ± 1.1	33.6 ± 1.2	>36/>36
Rspo4	>35	>35	>36/>36

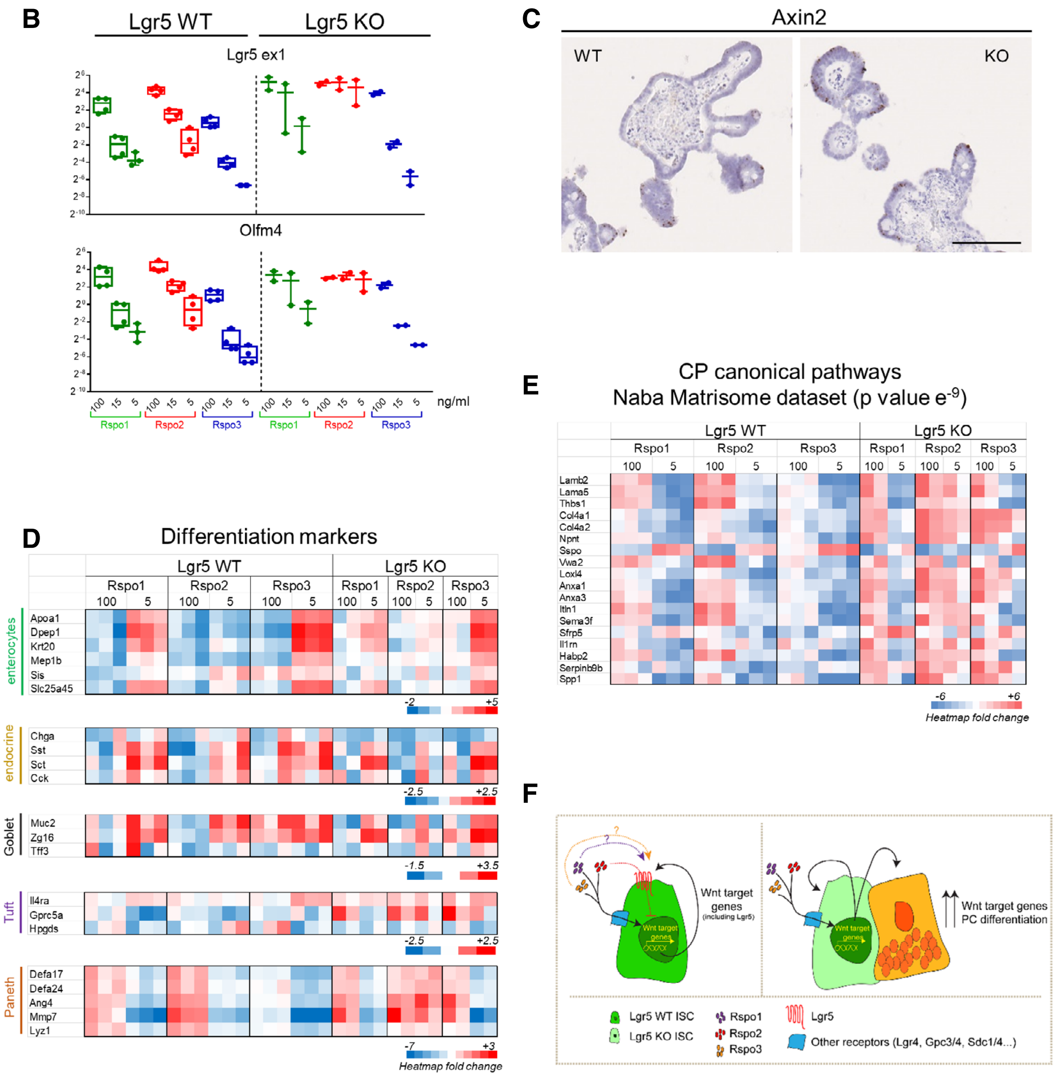

Figure EV5. Rspondin 2/Lgr5 interaction regulates stem cell fate in organoids.

A Expression levels of Rspondin ligands during intestinal maturation. Tables showing Ct values for *Rspo1*, *Rspo2*, *Rspo3*, and *Rspo4* genes expression levels analyzed by qRT–PCR on duodenum (Duo) and ileum (Ile) from E18.5 embryos (n = 11 samples originating from individual embryos) and adult animals (n = 3 samples originating from individual animals).

B Gene expression analysis by qRT–PCR of stem cell markers in Lgr5-DTREeGFP WT and KO organoids exposed to 100, 15, or 5 ng/ml Rspondins after 5 days of culture. Each symbol indicates the value for an organoid culture originated from a given embryo.

C Expression of the Wnt target gene *Axin2* by RNAscope in WT and KO organoids cultured in the presence of Rspondin 2 at 5 ng/ml. Scale bars: 100 µm.

D Heatmap showing the impact of Rspondin type and concentration on relative cell lineage differentiation (enterocyte, enteroendocrine, goblet, tuft, and Paneth cell lineages) in Lgr5-DTReGFP WT and KO organoids analyzed by RNAseq (n = 3 WT and 2 KO organoid culture samples, each originating from a given embryo).

E Heatmap showing the impact of Rspondin type and concentration on matrisome components gene expression (list from the Naba Matrisome dataset) in Lgr5-DTReGFP WT and KO organoids analyzed by RNAseq (n = 3 WT and 2 organoid culture samples, each originating from a given embryo).

F Scheme describing current hypothesis/model for Rspondins action on Wnt signaling regulation in ISC through Lgr5 and alternative receptors.
